# Recombinant *Escherichia coli* Strains with Inducible *Campylobacter jejuni* Single Domain Hemoglobin CHb Expression Exhibited Improved Cell Growth in Bioreactor Culture

**DOI:** 10.1371/journal.pone.0116503

**Published:** 2015-03-06

**Authors:** Li Xu, Wei Xiong, Jiang-Ke Yang, Jia Li, Xing-Wu Tao

**Affiliations:** College of Biology and Pharmaceutical Engineering, Wuhan Polytechnic University, Wuhan, China; Louisiana State University and A & M College, UNITED STATES

## Abstract

Maintaining an appropriate concentration of dissolved oxygen in aqueous solution is critical for efficient operation of a bioreactor, requiring sophisticated engineering design and a system of regulation to maximize oxygen transfer from the injected air bubbles to the cells. Bacterial hemoglobins are oxygen-binding proteins that transfer oxygen from the environment to metabolic processes and allow bacteria to grow even under microaerophilic conditions. To improve the oxygen utilization efficiency of cells and overcome the oxygen shortage in bioreactors, the gene coding for the *Campylobacter jejuni* single domain hemoglobin (CHb) gene was artificially synthesized and functionally expressed under the control of inducible expression promoters P_T7_ and P_vgh_ in *Escherichia coli*. The effects of the recombinants P_T7_-CHb and P_vgh_-CHb on cell growth were evaluated in aerobic shake flasks, anaerobic capped bottles and a 5-L bioreactor, and a pronounced improvement in cell biomass was observed for CHb-expressing cells. To determine the growth curves, CHb gene expression, and CHb oxygen-binding capacity of specific recombinants with different promoters, we determined the time course of CHb gene expression in the two recombinants by semi-quantitative RT-PCR and CO differential spectrum assays. Based on the growth patterns of the two recombinants in the bioreactor, we proposed different recombinant types with optimal performance under specific culture conditions.

## Introduction

To achieve efficient aerobic cultivation in a bioreactor, it is critical to maintain an appropriate concentration of dissolved oxygen in the broth. Due to the low mass transfer of oxygen from the gas bubbles to the aqueous solution, the content of dissolved oxygen usually becomes a significant limitation, especially in high cell density cultivation processes. To maximize the oxygen transfer from injected air bubbles to cells, sophisticated engineering designs and control systems are required, including the optimization of stirring and aeration and the addition of pure or enriched oxygen [1–3].

Hemoglobin (Hb) is a critical protein molecule involved in oxygen utilization in cells. As an iron-containing oxygen-transport metalloprotein, it carries oxygen from inner respiratory cells and delivers the oxygen as an input to the metabolic processes that provide energy and sustain multiple organismal functions. Although this oxygen-binding protein was first found in mammals, recent findings have indicated the almost ubiquitous presence of Hb in mammals, non-vertebrates, plants, and bacteria [4]. Bacterial hemoglobin is an oxygen-binding protein that allows bacteria to grow aerobically even under microaerophilic conditions. Three groups of hemoglobins have been identified in microorganisms thus far: single domain Hbs (sdHbs), flavohemoglobins (FHbs), and truncated Hbs (trHbs) [5]. *Vitreoscilla* hemoglobin (VHb), the first discovered bacterial hemoglobin, was isolated by Webster in 1966 from a *Vitreoscilla* species [6,7]. Recent studies demonstrated that the heterologous expression of the *Vitreoscilla* hemoglobin gene (*vgh*) substantially promoted both bacterial protein synthesis and oxygen diffusion, improving the aerobic metabolism of the host. This finding has intrigued the interest of scientists in the field of genetic engineering due to the potential of the heterologous expression of the *vgh* gene to improve industrial protein production systems by enhancing cell density, protein synthesis and oxidative metabolism, particularly under oxygen-limited conditions [5, 8–11].

Compared with the well-characterized VHb protein, few studies report the use of other bacterial hemoglobins in biotechnological applications. *Campylobacter jejuni* is a Gram-negative, foodborne pathogen. This microaerophilic organism grows optimally under conditions of 3 to 5% oxygen. Recent studies revealed that this microorganism contains two hemoglobin proteins, a single domain and a truncated hemoglobin [12, 13]. Studies on the single domain hemoglobin show that it is strongly and specifically induced after exposure to nitrosative stress and, unlike the archetypal single-domain globin Vgb, exhibits robust NO consumption and may contribute to the nitrosative stress resistance of *C. jejuni* [13]. Our preliminary experiments have shown that the expression of CHb could improve the growth of *E. coli* under microaerophilic conditions (data not shown), indicating that this CHb may be a useful tool for biotechnological applications.

In this study, we tried to develop a novel system based on the *C. jejuni* single domain hemoglobin gene CHb to improve the growth characteristics of cells under hypoxic cultivation conditions to resolve the conflict between the bacterial oxygen demand and the experimental oxygen supply capacity of the bioreactor. We first synthesized the CHb gene and then constructed four recombinants under the control of the inducible expression promoters P_T7_ and P_vgh_. We determined the growth curves of the recombinants in shake flasks, capped bottles and a 5-L bioreactor to evaluate the ability of CHb to improve cell growth under aerobic and anaerobic conditions, respectively. We further analyzed the expression time course of the CHb gene by semi-quantitative RT-PCR and CO differential spectrum assays to determine the relationship between promoter types, the level of CHb gene expression, oxygen-binding capacity and cell growth.

## Materials and Methods

### Synthesis of the *C. jejuni* trHb Gene

**Fig 1:**
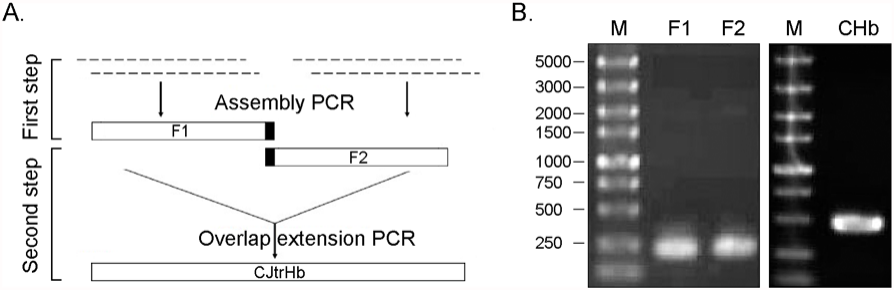
Synthesis of the CHb gene by a two-step gene synthesis method. A: Flow chart depicting the two-step CHb gene synthesis method; B: Agarose gels verifying the PCR product of assembly PCR and overlap extension PCR of the CHb gene.

This study used a two-step gene synthesis method [14] to synthesize the single domain Hb gene of *C. jejuni* (GenBank: KM007077). Briefly, a batch of adjacent oligonucleotides covering both DNA strands of the full length CHb gene was designed using Gene2Oligo software [15] and then synthesized by the solid-phase phosphoramidite method. The sequences of the oligonucleotides are listed in S1 Table. According to the two-step gene synthesis process described in Fig. 1, oligonucleotides were first assembled into two fragments with 15 overlapping bases by assembly PCR, and then these two fragments were assembled into the full length CHb gene by overlap extension PCR. The CHb gene was inserted into the pMD18-T vector (TaKaRa, Dalian, China) and verified by sequencing.

### Recombinant Plasmid Construction

**Fig 2:**
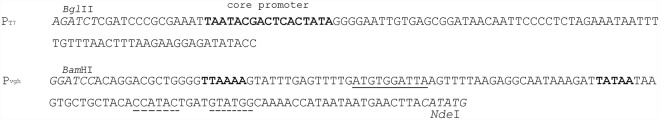
The nucleotide sequences of the promoters synthesized in this study. Italics indicate the restriction sites, and bolded letters are the conserved motifs of the promoters. Underlined nucleotides is a FNR-binding site, and dash line indicated is a portion of cAMP-CAP binding site.

The hypoxia-inducible promoter P_vgh_ of *Vitreoscilla* [16] was synthesized using the same process described above. The promoter sequences are listed in Fig. 2, and the oligonucleotides used to synthesize these promoters are listed in S2 Table. The IPTG-inducible expression recombinant P_T7_-CHb was constructed by inserting the CHb gene into the pET28a vector at the *Nde*I and *Xho*I sites and then transfected into *E. coli* BL21 (DE3) cells. Hypoxia-inducible recombinant P_vgh_-CHb was constructed by fusing P_vgh_ with the CHb gene by *Nde*I sticky ends to form P_vgh_-CHb fragments. These fragments were then inserted into the pUC18 vector at the *Bam*HI and *Xho*I sites and transfected into the *E. coli* DH5α strain.

### Determining the Growth Curves of the Recombinants in Flasks and Capped Bottles

To determine the growth curve of these recombinants in shake flasks, the *E. coli* strains were inoculated into a 500-mL volume flask with 100 mL of LB medium. For cells grown in capped bottles, 400-mL LB medium filled in a 500-mL volume capped bottle was deaerated with an ultrasonic cleaner and then sealed. Recombinant P_T7_-CHb was grown in medium containing 0.05 mM IPTG to induce CHb expression. All recombinants were inoculated into the medium at a 1/50 ratio and incubated at 37∞C in a thermostatic rotator (eccentric distance = 20 mm) with 180 rpm agitation rate. For every type of recombinant, three replicates were conducted. The optical density (OD_600_) of the cells were measured every 2 h. The specific growth rate (*μ*) of the recombinants was measured at logarithmic phase and calculated as the changes on the optimal density (OD) of the cell culture. The equation for cell specific growth rate was *μ* = 2.303(lg *OD*_*2*_-lg *OD*_*1*_)/(*t*_*2*_-*t*_*1*_). *OD*_*1*_ and *OD*_*2*_ indicated the cell optimal density value checked at the time point *t*_*1*_ and *t*_*2*_, respectively.

### Determination of the Growth Curve of the Recombinants in a Bioreactor

Batch cultivation of *E. coli* recombinants were performed in a 5-L bioreactor (BIOSTAT, Sartorius AG) under controlled temperature and dissolved oxygen levels. The recombinant cells were inoculated into the medium at a ratio of 1/100, and the OD_600_ value of the broth was measured every 2 h. Approximately 50 mL of broth was sampled from the bioreactor and the cells were collected by centrifuging with 5,000 g centrifugal force for 5 min to measure the wet cell weight. A 10 mL aliquot of broth was also collected, and then the cells were subjected to total RNA extraction.

### Semi-Quantitative RT-PCR Assay to Determine CHb Expression

The total RNA of *E. coli* cells was extracted using a Trizol RNA isolation kit (Invitrogen Life Technologies) according to the manufacturer’s instructions. The first strand of cDNA was synthesized by reverse transcription using a RevertAid First Strand cDNA Synthesis Kit (Fermentas). Primers SemiCJF (5’-GATTGTGTGCCTATTTTGC-3’) and SemiCJR (5’-CTATATCGATATAAGATTTAGC-3’) were used to amplify a 300-bp CHb fragment in a semi-quantitative RT-PCR assay to determine CHb gene expression. The *E. coli* house-keeping gene glyceraldehyde-3-phosphate dehydrogenase (GAPDH) was used as an internal standard, and the primer pair GAPDHF (5’-GTGGTTATGACTGGTCCGTC-3’) and GAPDHR (5’-GTCGTTCAGAGCGATACCAGC-3’) was used to amplify a 520-bp GAPDH fragment. Every 50-μL PCR reaction mixture contained 1×buffer, 200 mM dNTPs, 10 pmol of each primer, 50 ng of reverse transcription products and 2 U of Pfu Turbo DNA polymerase (Stratagene, La Jolla, CA). PCR amplification was conducted in a thermal cycler (Biometra, Germany) with an initial denaturation at 94 ∞C for 5 min; 20 cycles of 94 ∞C for 50 s, 53 ∞C for 50 s, and 72 ∞C for 50 s; and a final 6 min extension at 72 ∞C.

The PCR products were resolved by electrophoresis through a 1.5% agarose gel and recorded by a fluorescence scanner (Bio-rad ChemiDoc System). Band volumes were quantified with ImageQuaNT software (Molecular Dynamics). CHb and GAPDH cDNA PCR product ratios were calculated and then normalized with GAPDH counts to obtain CHb expression profiles. The levels of gene expression are reported as relative intensity vs. GAPDH.

### CO Difference Spectrum Method to Determine Hemoglobin Activity

Approximately 200 mL of recombinant cells were centrifuged, and the cell pellet was re-suspended in assay buffer (10 mM Tris-HCl, pH 8.0, 200 mM NaCl) and lysed by sonication. The total protein concentration of the lysates was determined by the Bradford method [17]. The reductant sodium hydrosulfite powder was added to the cell lysates to a final concentration of 50 mg/mL and then incubated on ice for 20 min. Carbon monoxide gas was bubbled through the protein liquid for ten minutes followed by incubation in the dark for ten minutes. The spectroscopic differences between CO-treated samples and the control were scanned from 400 nm to 600 nm in a UV-vis spectrophotometer (DU-640, Beckman).

## Results

### Growth Curves of the Recombinants in Shake Flasks

**Fig 3:**
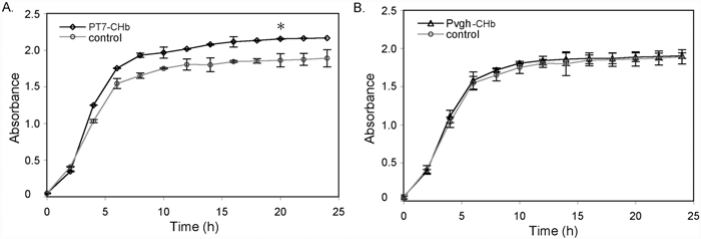
Growth curves of the recombinants P_T7_-CHb, P_vgh_-CHb and the controls in flask culture. *E. coli* cells carrying blank vectors were used as the controls. A: The recombinant P_T7_-CHb; B: The recombinant P_vgh_-CHb. Seed cells of the recombinants were inoculated into a 500-mL volume flask with 100 mL of LB medium containing 50 mg/ml ampicillin for P_vgh_-CHb and 20 mg/mL tetracycline for P_T7_-CHb at a 1/50 ratio, and incubated at 37∞C on a thermostatic rotator (eccentric distance = 20 mm) with 200 rpm agitation rate. The optical density (OD_600_) of the cells were measured every 2 h. The error bars indicated the standard deviation generated from three replicates data. * indicates the significant differecne (P<0.05) at 20 h time point calculated by one-way ANOVA analysis.

We first determined the growth curves of the four recombinants (P_T7_-CHb, P_vgh_-CHb and their empty vector controls) in aerobic shake flask cultures. Recombinant P_T7_-CHb clearly promoted the growth of *E. coli* cells under aerobic conditions. As shown in Fig. 3A, the IPTG-inducible recombinant P_T7_-CHb grew more rapidly than the control, with the cell density of the recombinant reaching OD = 2.2 in the stationary phase, 22.2% higher than the control (Fig. 3A). And the specific growth rate of recombinant P_T7_-CHb in the logarithmic phase reached *μ*_*T7*_ = 0.448, which apparently higher than the control (*μ*_*T7-control*_ = 0.339). When the CHb gene was under the control of the oxygen-dependent *Vitreoscilla* hemoglobin promoter P_vgh_, which is specifically induced by low dissolved oxygen levels [16], the P_vgh_-CHb recombinant did not grow differently than the control (Fig. 3B).

### Growth Curves of the Recombinants in Capped Bottles

**Fig 4:**
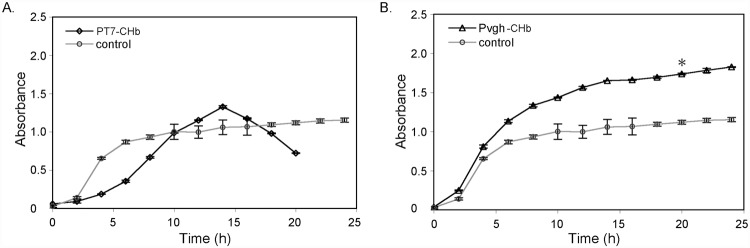
Growth curves of the recombinants P_T7_-CHb and P_vgh_-CHb and the corresponding empty vector controls in anaerobic capped bottles. A: The recombinant P_T7_-CHb; B: The recombinant P_vgh_-CHb. LB medium containing 50 mg/ml ampicillin for Pvgh-CHb and 20 mg/mL tetracycline for P_T7_-CHb was deaerated with an ultrasonic cleaner for 20 min. *E. coli* cells carrying the blank vector were used as controls. Seed cells of the recombinants were inoculated into the 500-mL volume capped bottle filled with 400-mL LB medium at a ratio of 1/50 and cultured at 37 ∞C in a thermostatic rotator (eccentric distance = 20 mm) with 200 rpm agitation rate. For recombinant PT7-CHb, 0.05 mM IPTG was added to the medium to induce CHb expression. The optical density (OD_600_) of the cells were measured every 2 h. The error bars indicated the standard deviation generated from three replicates data. * indicates the significant differecne (P<0.05) at 20 h time point calculated by one-way ANOVA analysis.

We next determined the growth curves of these recombinants in capped bottles, which provide a relatively low oxygen or anaerobic environment (Fig. 4). Low oxygen conditions strongly inhibit the growth of *E. coli* cells. As shown by the control curve (without the CHb gene), the optimal density of the cells in the stationary phase was approximately OD_600_ = 1.1 (Fig. 4), far lower than that in the flask culture (Fig. 3). Meanwhile, under anaerobic conditions, recombinant P_vgh_-CHb grew at a significantly higher rate than the control (Fig. 4B). As shown in Fig. 4B, the recombinant P_vgh_-CHb grew more rapidly than the control, with the cell density of the recombinant reaching OD = 1.75 in the stationary phase, 45.8% higher than the control. And the specific growth rate of recombinant P_vgh_-CHb in the logarithmic phase reached *μ*_*vgh*_ = 0.287, which apparently higher than the control (*μ*_*vgh-control*_ = 0.225). This increased growth could result from the anaerobic or limited oxygen condition inducing the promoter P_vgh_ to initiate CHb expression, thus enhancing the ability of cells to capture oxygen and sustain cell growth under anaerobic conditions. The sharply drop down on cell density was observed in recombinant P_T7_-CHb in the later part of the logarithmic phase. This led the growth curve to decrease from the highest OD_600_ = 1.4 at the later part of the logarithmic phase to OD_600_ = 0.75 at 20 h (Fig. 4A).

### Growth Curves in Bioreactors

**Fig 5:**
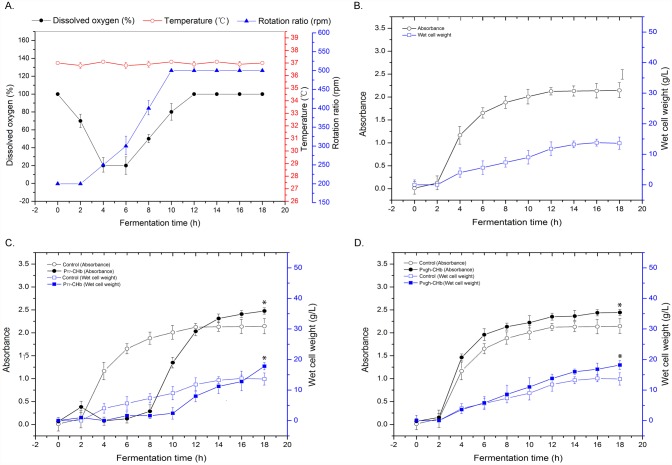
Cultivation parameters and growth conditions of the recombinant cells in the 5-L bioreactor. A: The cultivation parameters; B: The control cells carrying the empty pUC18 vector; C: The recombinant P_T7_-CHb; D: The recombinant P_vgh_-CHb. *E. coli* cells carrying blank vectors were used as the controls. Seed cells of the recombinants were inoculated at a 1:100 ratio into LB medium containing 50 mg/ml ampicillin for P_vgh_-CHb and 20 mg/mL tetracycline for P_T7_-CHb. For recombinant P_T7_-CHb, 0.05 mM IPTG was added into the medium to induce CHb expression. The culture temperature was kept at 37 ∞C, and the pH value of the medium was kept at pH 6.8 with 200 mM NaOH titration. Before inoculation, LB liquid was injected into sterile air for 30 min, and then the dissolved oxygen (DO) of the medium was set as 100%. The error bars indicated the standard deviation generated from three replicates data. * indicates the significant differecne (P<0.05) at 18 h time point calculated by one-way ANOVA analysis.

To test whether the recombinant *E. coli* expressing hemoglobin CHb could improve the cell growth in the bioreactor or not, we recorded the growth curves of four recombinants in a 5-L bench top bioreactor (Fig. 5). Due to better temperature, pH and aeration control (Fig. 5A), the recombinant cells grown better in bioreactor than in the flasks and capped bottles. As shown by the control cells, the cell density reached and the wet cell weight reached OD_600_ = 2.2 and 14.0 g/L, respectively, apparently higher than those in the flasks (Fig. 3) and capped bottles (Fig. 4).

The growth curve of the IPTG-inducible expression recombinant P_T7_-CHb showed that the lag phase of the cell was significantly elongated under IPTG induced condition, which made the lag phase of the culture elongated by approximately 8 h and then entered into the logarithmic phase. Despite the elongation on cell growth, CHb still promoted the cell growth of the recombinant strain in the bioreactor. When the culture reached the stationary phase at approximately 18 h, the cell density reached OD_600_ = 2.5, 16.3% higher than that of the control, and the wet cell weight was 17.5 g/L, 25.0% higher than that of the control (Fig. 5B and 5C).

Although P_vgh_ was regarded as a hypoxia-inducible promoter that would only respond to oxygen-limited conditions [16], such as those in a capped bottle (Fig. 3 and Fig. 4), P_vgh_-CHb recombinants still exhibited an improved cell growth rate and final biomass. At the stationary phase (18 h time point), the cell density reached OD_600_ = 2.4, and the wet cell weight reached 18.0 g/L, 14.3% and 28.6% higher than those of the control cells, respectively (Fig. 5D). And the specific growth rate of recombinant P_vgh_-CHb in the logarithmic phase reached *μ*_*vgh*_ = 0.287, which apparently higher than the control (*μ*_*vgh-control*_ = 0.225).

### Semi-Quantitative RT-PCR Assay to Monitor the Expression of CHb in Bioreactor Culture

**Fig 6:**
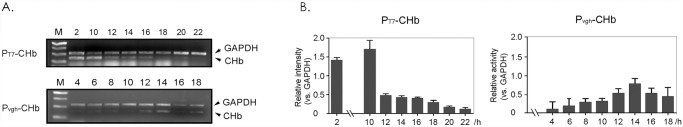
Semi-quantitative RT-PCR assays of the levels of CHb transcription in four recombinants in the bioreactor. A: Time course of CHb expression visualized by agarose gels with GAPDH used as an internal control; B: The signal intensities of each band were quantified and normalized to those of the corresponding GAPHD band and reported as relative values.

Semi-quantitative RT-PCR was conducted to determine the expression level of the CHb gene under the control of the two promoters. As shown in Fig. 6, the time course expression analysis revealed the constitutive and inducible expression characteristics of the CHb recombinants. For the IPTG inducible expression recombinant P_T7_-CHb, the highest level of CHb expression was observed in the lag phase and the early logarithmic phase (from 0 h to 10 h). At that point, the relative density of CHb was approximately 1.7-fold greater than that of GAPDH (10 h time point). After that point, the cells entered into the middle stage of the logarithmic phase and then the stationary phase, and the expression level of CHb gradually decreased (Fig. 6A and 6B).

Unexpectedly, the expression of CHb controlled by the oxygen-dependent promoter P_vgh_ was still detected by RT-PCR in the early stage of the cultivation, when the DO remained high. Later, as the DO decreased, the expression level of CHb gradually increased to 0.75-fold that of GAPDH at the 14 h time point, when the cells began to enter into the stationary phase. After 14 h time point, the mRNA level of CHb gradually declined in the stationary phase as the aeration and DO increased (Fig. 6A and 6B).

### CO Differential Spectrum Assays to Monitor the Activity of CHb in Bioreactor Culture

**Fig 7:**
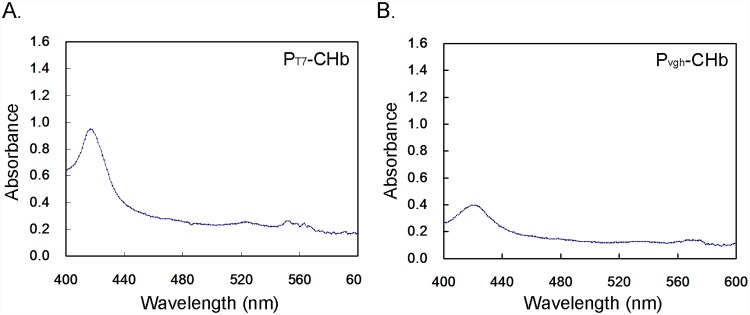
CO differential spectrum assays of the CHb in four recombinant cultures in the bioreactor. A: The recombinant P_T7_-CHb; B: The recombinant P_vgh_-CHb.

As the expression of CHb at the transcriptional level may not coincide with protein activity, CO differential spectrum assays were conducted to determine the CHb hemoglobin of each recombinant. The spectrogram scan from 400 nm to 600 nm exhibited an intense absorption peak at approximately 419 nm, with two secondary peaks at approximately 520–560 nm (Fig. 7). To compare the hemoglobin activity of the recombinants, we compared the intensity of the 419-nm peaks among recombinants. The highest hemoglobin activity was observed in recombinant P_T7_-CHb, with an absorbance value OD = 1.42. The fact that the P_T7_-CHb strain exhibits the highest hemoglobin activity explains why the cell density and wet cell weight were still higher than those of the control in the stationary phase (Figs. 3, 4, 5), although cell autolysis and growth inhibition occurred in the lag phase and the stationary phase (Figs. 4, 5). We determined that the over-expression of CHb by the P_T7_-CHb recombinant may represent a heavy burden for cell growth, as commonly occurs in P_T7_-regulated recombinant expression hosts [18].

## Discussion

Three types of hemoglobin have been identified in bacteria thus far, single domain hemoglobins (sdHbs), flavohemoglobins (FHbs) and truncated hemoglobins (trHbs) [5]. As revealed by the first and most thoroughly studied bacterial hemoglobins *Vitreoscilla* hemoglobin (VHb), bacterial hemoglobin can bind to oxygen, particularly at low concentrations, and carry and deliver that oxygen to the terminal respiratory oxidase to enhance ATP production under hypoxic conditions [19–21]. VHb expression in heterologous hosts often enhances cell density, oxidative metabolism, and engineered expression products, especially under oxygen-limiting conditions [8–11]. In contrast with the well-characterized VHb protein, other bacterial hemoglobins have not been employed in biotechnological applications. This study reports the development of a novel system based on the functional expression of the *C. jejuni* single domain hemoglobin CHb to improve the oxygen utilization efficiency of cells in a bioreactor.

The inducible promoters P_T7_ and P_vgh_ were used to control the expression of CHb, forming two types of recombinants. The growth curves of the two recombinants determined under aerobic (in flask), microaerobic (capped bottle) and bioreactor conditions demonstrated that CHb increased the cell density and biomass (Figs. 3, 4, 5). As detected by semi-quantitative RT-PCR and CO differential spectrum assays, this improvement may be due to the efficient expression of CHb and to the hemoglobin activity of CHb in the cells (Fig. 6 and Fig. 7).

As observed in aerobic flasks and microaerobic capped-bottles, recombinant P_vgh_-CHb only improved cell growth under capped-bottle conditions, possibly because the oxygen dependent promoter P_vgh_ was modulated by the involvement of FNR-like or Crp proteins and was only activated under oxygen limiting conditions [16, 22, 23]. Surprisingly, the recombinant P_vgh_-CHb was even expressed in the bioreactor when the dissolved oxygen value remained at a high level (Fig. 6).

As frequently reported, the expression of *Vitreoscilla* hemoglobin gene was natively controlled by the oxygen-dependent promoter (P_vgh_), and was maximally activated both in *Vitreoscilla* and *E. coli* under microaerobic conditions [16, 22–25]. We also detected that when the disolved oxygen declined in the bioreactor, the expression level of CHb increased correspondingly (Fig. 5 and Fig. 6). Mechanistic analysis found the expression of *vgh* gene was positively controlled by the transcriptional activator FNR [16]. As revealed by Fig. 2, an FNR binding site exists within the promoter sequence (Fig. 2). FNR protein of *E. coli* is an oxygen—responsive transcriptional regulator. The *fnr* gene was expressed under both aerobic and anaerobic conditions and was subject to autoregulation [25].

As revealed by reduced expression levels in *cya* mutant *E. coli* strains, P_vgh_ was also positively controlled by cAMP-CAP complex, and thus responsed to the cAMP or glucose level in the cells [16]. As shown by Fig. 2, a CAP binding site was positioned within the P_vgh_ promoter sequence. Recent reports by Pablos et al [26] also indicated the expression of *vgh* gene could increase the efficiency of aerobic metabolism by enhancing the consumption of NADH in the respiratory chain, leading to increased activity of the tricarboxylic acid (TCA) cycle. This would cause a higher TCA activity and reduced acetate formation resulted in a significant increase in growth and glucose consumption rates. Furthermore, the P_vgh_-controlled expression of VHb was also induced by nitrosative, and protected the heterologous host from nitrosative stree [12, 24].

Considering the complex regulation mechanisms on promoter P_vgh_, we speculated that while the DO remained high in the bioreactor, zones of hypoxia might exist in bioreactor tank due to defaults in the engineering design [2, 3, 27]. When the culture reached the logarithmic phase, the DO of the whole tank rapidly declined with the rapid cell growth and induced high levels of P_vgh_-CHb expression, and in the later part of the logarithmic phase, the expression of CHb accumulated to the maximal level (Fig. 5A). Moreover, as reported by Khosla et al, although VHb was significantly induced under microaerobic conditions, there still existed low-level expression of VHb under aerobic condition [16], which funding is same as our study (Fig. 6).

The cell growth delay was observed on recombinant P_T7_-CHb in this study. Especially in the bioreactor, the lag phase was elongated to 8 h time point (Fig. 5C). The IPTG-inducible phage T7 late promoter P_T7_ is regarded as a strong promoter and is broadly used for the over-expression of heterologous genes in *E. coli* [18, 28]. The P_T7_-CHb recombinant exhibited higher CHb transcription and a higher CO differential spectrum than P_vgh_-CHb (Fig. 6 and Fig. 7), potentially representing a heavy burden on the physiological processes and growth of the cells, leading to stress reactions and retarded cell growth. To adapt P_T7_-CHb cells to the bioreactor cultivation conditions, strategies such as reduction the concentration or batch-fed the IPTG may be needed.

While the microaerobic-inducible recombinant P_vgh_-CHb exhibited lower CHb expression and hemoglobin activity, the cell density and biomass were still significantly improved. This appropriate CHb expression level did not impose a physiological burden on the cells, allowing for healthy cell growth (Figs. 4 and 5). Moreover, in contrast with the P_T7_-CHb recombinant, CHb expression in the P_vgh_-CHb recombinant was regulated by the dissolved oxygen in the bioreactor broth. When the DO declined, inhibiting cell growth, the CHb was expressed, enhancing the cells’ ability to capture oxygen, compensating for the lack of dissolved oxygen. Based on these results, P_vgh_-CHb appears to be an excellent system for bioengineering.

In summary, *Campylobacter jejuni* single domain hemoglobin CHb can increase the cell density and biomass in bioreactor cultures. The system developed in this study could be used to overcome the inability of the bioreactor to supply sufficient oxygen to meet bacterial demand. Recombinant P_T7_-CHb exhibited significant improvements in cell density and biomass, while serious cells growth delay was observed in the bioreactor due to CHb over-expression. Although P_vgh_ leads to a lower level of CHb expression in the bioreactor, CHb still improved the cell density and wet cell weight. The hypoxia-inducible feature of this expression system resulted in the autoregulation of CHb expression according to the dissolved oxygen level. Therefore, this system is highly suitable for applications in large-scale cultivation.

## Supporting Information

S1 Table Oligonucleotides for synthesis the CHb gene. (DOC)

S2 Table Oligonucleotides for synthesis the promoters Pvgh promoter. (DOC)
