# Supplementary material for: Recombinant Escherichia coli Strains with Inducible Campylobacter jejuni Single Domain Hemoglobin CHb Expression Exhibited Improved Cell Growth in Bioreactor Culture
Source: PLoS One. 2015 Mar 6;10(3):e0116503. doi: 10.1371/journal.pone.0116503 (PMC4352031; doi:10.1371/journal.pone.0116503)
Supplement: S1 Table — (DOC) [file pone.0116503.s001.doc]

Table S1 Oligonucleotides for synthesis the CHb gene

| ID | Nucleotide sequence |
| --- | --- |
| CJR0 | AATTTGTTCTTTTGTCATATG |
| CJF0 | CATATGACAAAAGAACAAATTCAAATCATCAAAGATTGT |
| CJR21 | TTTGCAAAATAGGCACACAATCTTTGATGATTTG |
| CJF39 | GTGCCTATTTTGCAAAAAAATGGAGAGGATTTAA |
| CJR55 | ATTATTTTATAAAACTCATTGGTTAAATCCTCTCCATTTT |
| CJF73 | CCAATGAGTTTTATAAAATAATGTTTAATGATTATCCTGAG |
| CJR95 | CATATTAAACATAGGTTTTACCTCAGGATAATCATTAAAC |
| CJF114 | GTAAAACCTATGTTTAATATGGAAAAACAAATTTCAGGA |
| CJR135 | AAGCTTTTGGTTGTTCTCCTGAAATTTGTTTTTC |
| CJF153 | GAACAACCAAAAGCTTTAGCAATGGCGATTTT |
| CJR169 | ATTTTTAGCCGCCATTAAAATCGCCATTGCTA |
| CJF185 | AATGGCGGCTAAAAATATAGAAAATTTGGAAAATATG |
| CJR201 | TTATCAACAAAGCTTCTCATATTTTCCAAATTTTCTAT |
| CJF222 | AGAAGCTTTGTTGATAAAGTTGCCATAACTCAT |
| CJR239 | TCTTTAACTCCTAAATTAACATGAGTTATGGCAACT |
| CJF255 | GTTAATTTAGGAGTTAAAGAAGAGCATTATCCTATAGT |
| CJR275 | TAAAAGGCAAGCTCCAACTATAGGATAATGCTCT |
| CJF293 | TGGAGCTTGCCTTTTAAAGGCTATTAAAAATCTTTT |
| CJR309 | GGCTTCATCAGGATTTAAAAGATTTTTAATAGCCTT |
| CJF329 | AAATCCTGATGAAGCCACTCTTAAAGCTTGGG |
| CJR345 | TTTTCCATAAGCAACTTCCCAAGCTTTAAGAGT |
| CJF361 | AAGTTGCTTATGGAAAAATTGCTAAATTTTATATCGA |
| CJR378 | CATAGAGCTTTTTTTCTATATCGATATAAAATTTAGCAAT |
| CJF398 | TATAGAAAAAAAGCTCTATGATAAATAACTCGAGgatg |
| CJR418 | cctaactaactgtgcacatcCTCGAGTTATTTAT |
| CJR436 | tgcacagttagttagg |
