# Supplementary material for: Recombinant Escherichia coli Strains with Inducible Campylobacter jejuni Single Domain Hemoglobin CHb Expression Exhibited Improved Cell Growth in Bioreactor Culture
Source: PLoS One. 2015 Mar 6;10(3):e0116503. doi: 10.1371/journal.pone.0116503 (PMC4352031; doi:10.1371/journal.pone.0116503)
Supplement: S2 Table — (DOC) [file pone.0116503.s002.doc]

Table S2 Oligonucleotides for synthesis the promoters P_vgh_ promoter

| ID | Nucleotide sequence |
| --- | --- |
| PvghR0 | GTCCTGTGGATCCgtc |
| PvghF0 | gacGGATCCACAGGACGCTGGGGTTAAAAGTA |
| PvghR16 | CCACATCAAAACTCAAATACTTTTAACCCCAGC |
| PvghF32 | TTTGAGTTTTGATGTGGATTAAGTTTTAAGAGGCA |
| PvghR49 | CAGCACTTATTATAATCTTTATTGCCTCTTAAAACTTAAT |
| PvghF67 | ATAAAGATTATAATAAGTGCTGCTACACCATACTGATGT |
| PvghR89 | TTATTATGGTTTTGCCATACATCAGTATGGTGTAG |
| PvghF106 | ATGGCAAAACCATAATAATGAACTTACATATGgaaga |
| PvghR124 | gcctgatttgttcccttcttcCATATGTAAGTTCA |
| PvghR143 | agggaacaaatcaggc |
